# Supplementary material for: Psychological and Social Impact of HIV on Women Living with HIV and Their Families in Low- and Middle-Income Asian Countries: A Systematic Search and Critical Review
Source: Int J Environ Res Public Health. 2022 May 30;19(11):6668. doi: 10.3390/ijerph19116668 (PMC9180788; doi:10.3390/ijerph19116668)
Supplement: Supplementary file 1 [file ijerph-19-06668-s001.zip › ijerph-1706830-supplementary.pdf]

Supplementary Materials S1: Full searching strategy by databases

Database(s): **Ovid Emcare**
Search Strategy:

#	Searches	Results
1	human immunodeficiency virus/	21298
2	acquired immune deficiency syndrome/	77403
3	(HIV* or "Human immunodeficiency virus" or AIDS).tw,kw.	438274
4	1 or 2 or 3	452954
5	female/ or marriage/ or spouse/ or wife/	9399966
6	mother/ or adolescent mother/ or expectant mother/ or mother child relation/	66053
7	female/ or female by marital status/ or girl/	9394650
8	(Wives or Wife or Mothers or female* or girl* or wom?n).tw,kw.	2455822
9	5 or 6 or 7 or 8	9818864
10	risk factor/	917105
11	sexual behavior/ or casual sex/ or concurrent sexual partnership/ or contraceptive behavior/ or extramarital sex/ or hiv serosorting/ or premarital sex/ or prostitution/ or sexual practice/ or sexual promiscuity/ or sexual violence/ or transactional sex/ or unsafe sex/	86054
12	condom/	10909
13	social aspect/	25625
14	peer pressure/	768
15	social norm/	1805
16	cultural factor/	23440
17	socioeconomics/ or economic aspect/ or educational status/ or lowest income group/ or poverty/	95665
18	social interaction/	1124
19	social environment/ or environmental factor/	44076
20	social stigma/ or stigma/	11233
21	social discrimination/ or social problem/ or employment discrimination/ or social distance/	9750
22	psychological aspect/	53200
23	health/ or child health/ or family health/ or mental health/ or sexual health/ or women's health/	133489
24	school attendance/ or absenteeism/	9595
25	health care access/	31015
26	(predictor* or "risk factor*" or determinant* or "sexual behaviour" or "multiple sex partner*" or "sell* sex*" or extramarital* or "transactional sex" or prostitut* or "sex work" or condom* or "unsafe sex" or "unprotected sex" or knowledge or "social influenc*" or "peer influenc*" or "social norm" or cultur* or sociocultural* or socioeconomic* or "social environmental*" or	4647253

	socioenvironment* or Stigma or discriminat* or "psychological impact" or "social impact" or "psychosocial impact" or stress or distress or depression).tw,kw.	
27	or/10-26	5322037
28	family/	81842
29	(family* or families).tw,kw.	1070817
30	28 or 29	1100542
31	developing country/	79007
32	((Developing or Less developed or low resource* or disadvantaged or resource limited or poor or low* or middle income*) adj (countr* or region* or nation? or area*)).tw,kw.	89188
33	31 or 32	144881
34	4 and 9 and 27 and 30 and 33	841
35	limit 34 to yr="2004 - 2021"	183

Database(s): **CINAHL**
Search Strategy:

#	Searches	Results
S1	(MH "HIV-Infected Patients") OR (MH "Human Immunodeficiency Virus") OR (MH "HIV-1")	10,938
S2	TI ((HIV* or "Human immunodeficiency virus" or AIDS)) OR AB ((HIV* or "Human immunodeficiency virus" or AIDS))	129,961
S3	(MH "Acquired Immunodeficiency Syndrome") OR (MH "AIDS Patients")	19,582
S4	S1 OR S2 OR S3	135,254
S5	(MH "Female")	2,132,233
S6	(MH "Married Women") OR (MH "Single Women") OR (MH "Women")	22,887
S7	(MH "Mothers") OR (MH "Expectant Mothers") OR (MH "Adolescent Mothers") OR (MH "Surrogate Mothers")	46,888
S8	TI ((Wives or Wife or Mothers or female* or girl* or wom?n)) OR AB ((Wives or Wife or Mothers or female* or girl* or wom?n))	749,596
S9	S5 OR S6 OR S7 OR S8	2,303,417
S10	(MH "Sexual Abuse")	9,465
S11	(MH "Unsafe Sex")	3,481
S12	(MH "Sexual Harassment")	2,227
S13	(MH "Bisexuality")	1,492
S14	(MH "Heterosexuality")	2,073
S15	(MH "Risk Factors")	196,278
S16	(MH "Sexual Partners")	9,882

S17	(MH "Prostitution") OR (MH "Unsafe Sex")	3,481
S18	(MH "Condoms")	8,161
S19	(MH "Socioeconomic Factors") OR (MH "Illiteracy") OR (MH "Poverty") OR (MH "Educational Status") OR (MH "Employment") OR (MH "Unemployment") OR (MH "Social Norms") OR (MH "Social Networks")	193,503
S20	(MH "Stigma")	18,823
S21	(MH "Discrimination")	12,231
S22	(MH "Psychosocial Aspects of Illness") OR (MH "Morals") OR (MH "Social Isolation")	22,863
S23	(MH "Stress") OR (MH "Stress, Psychological")	66,095
S24	(MH "Depression")	120,367
S25	(MH "Income")	22,095
S26	(MH "Adolescent Nutrition") OR (MH "Child Nutrition") OR (MH "Nutritional Status") OR (MH "Infant Nutrition")	27,865
S27	(MH "Adolescent Health") OR (MH "Child Health") OR (MH "Family Health") OR (MH "Health Status") OR (MH "Mental Health") OR (MH "Wellness")	135,966
S28	(MH "Health Services Accessibility")	97,272
S29	(MH "Absenteeism")	5,033
S30	(MH "Psychological Well-Being")	30,301
S31	TI ((predictor* or "risk factor*" or determinant* or "sexual behaviour" or "multiple sex partner*" or "sell* sex*" or extramarital* or "transactional sex" or prostitut* or "sex work" or condom* or "unsafe sex" or "unprotected sex" or knowledge or "social influenc*" or "peer influenc*" or "social norm" or cultur* or sociocultural* or socioeconomic* or "social environmental*" or socioenvironment* or Stigma or discriminat* or "psychological impact" or "social impact" or education or "school attendance" or "withdraw* from school" or "psychosocial impact" or stress or distress or depression or employment or "loss of job" or Income or "nutrition security" or "food insecurity" or health or "physical health" or wellbeing or "Healthcare accessibility*" or Absenteeism or religio* or Consequence*)) OR AB ((predictor* or "risk factor*" or determinant* or "sexual behaviour" or "multiple sex partner*" or "sell* sex*" or extramarital* or "transactional sex" or prostitut* or "sex work" or condom* or "unsafe sex" or "unprotected sex" or knowledge or "social influenc*" or "peer influenc*" or "social norm" or cultur* or sociocultural* or socioeconomic* or "social environmental*" or socioenvironment* or Stigma or discriminat* or "psychological impact" or "social impact" or "psychosocial impact" or stress or distress or depression))	1,549,822
S32	S10 OR S11 OR S12 OR S13 OR S14 OR S15 OR S16 OR S17 OR S18 OR S19 OR S20 OR S21 OR S22 OR S23 OR S24 OR S25 OR S26 OR S27 OR S28 OR S29 OR S30 OR S31	1,936,189
S33	(MH "Family")	44,723
S34	TI ((family* or families)) OR AB ((family* or families))	272,246
S35	S33 OR S34	287,870
S36	(MH "Developing Countries") OR (MH "Indian Ocean Islands") OR (MH "Low and Middle Income Countries") OR (MH "Asia")	28,756

S37	TI (((Developing or Less developed or low resource* or disadvantaged or resource limited or poor or low* income*) N (countr* or region* or nation? or area*))) OR AB (((Developing or Less developed or low resource* or disadvantaged or resource limited or poor or low* or middle income*) N (countr* or region* or nation? or area*)))	97
S38	S36 OR S37	28,818
S39	S4 AND S9 AND S32 AND S35 AND S38 (Published Date: 20040101-20221231)	47

Database(s): **PsycINFO**
Search Strategy:

#	Searches	Results
1	hiv/ or "aids (attitudes toward)"/ or aids prevention/ or *HIV Testing/	43172
2	(HIV* or "Human immunodeficiency virus" or AIDS).ti,ab,id.	75092
3	1 or 2	75333
4	wives/ or human females/ or spouses/	109509
5	exp EXPECTANT MOTHERS/ or exp UNWED MOTHERS/ or exp ADOLESCENT MOTHERS/ or exp SINGLE MOTHERS/ or exp MOTHERS/	44768
6	(Wives or Wife or Mothers or female* or girl* or wom?n).ti,ab,id.	746674
7	4 or 5 or 6	759753
8	exp Condoms/ or exp Sexual Risk Taking/ or exp Sexual Partners/	15923
9	exp Sexual Partners/	5115
10	risk factors/ or psychosocial factors/	125029
11	socioeconomic status/ or family socioeconomic level/ or income level/ or lower class/ or social class/ or disadvantaged/ or economic security/ or "income (economic)"/ or poverty/	62724
12	exp Sociocultural Factors/	126837
13	exp Emotional Trauma/ or exp Stress/ or exp Distress/ or exp Psychological Stress/ or exp Well Being/	211167
14	major depression/	137841
15	exp DISCRIMINATION/ or exp SOCIAL DISCRIMINATION/	52937
16	stigma/	15171
17	(predictor* or "risk factor*" or determinant* or "sexual behaviour" or condom* or "unsafe sex" or "unprotected sex" or "transactional sex" or prostitute* or "social influence" or "social norm" or cultur* or "sociocultural factor*" or socioeconomic* or "social environmental*" or socioenvironment* or Stigma or discrimination or "Psychological impact" or "Social impact" or Education or "Psychosocial impact" or stress or distress or depression).ti,ab,id.	1501319
18	or/8-17	1662981

19	family conflict/ or mother-child relations/ or parenting/	37831
20	*family/	33154
21	(family* or families).ti,ab,id.	407965
22	19 or 20 or 21	432552
23	3 and 7 and 18 and 22	1799
24	exp Developing Countries/	6015
25	((Developing or Less developed or low resource* or disadvantaged or resource limited or poor or low* or middle income*) adj (countr* or region* or nation? or area*)).ti,ab,id.	17343
26	24 or 25	18829
27	23 and 26	53

Database(s): **Ovid MEDLINE(R)**
Search Strategy:

#	Searches	Results
1	exp HIV/ or HIV-1/ or HIV-2/ or HIV INFECTIONS/	260283
2	Acquired Immunodeficiency Syndrome/	77403
3	(HIV* or "Human immunodeficiency virus" or AIDS).tw,kf.	439882
4	or/1-3	474973
5	mothers/ or surrogate mothers/	52441
6	Female/	9391282
7	Women/	15138
8	(Wives or Wife or Mothers or female* or girl* or wom?n).tw,kf.	2463234
9	or/5-8	9812155
10	sexual behavior/ or extramarital relations/ or hiv serosorting/ or sex work/ or safe sex/ or sexual harassment/ or bisexuality/ or heterosexuality/ or homosexuality/ or transsexualism/ or unsafe sex/	91745
11	Condoms/	10909
12	Sexual Partners/	19299
13	culture/ or social environment/ or social isolation/ or social norms/ or socioeconomic factors/ or poverty/ or poverty areas/ or social class/	321694
14	SOCIAL STIGMA/	11233
15	"Discrimination (Psychology)"/	19915
16	STRESS, PSYCHOLOGICAL/	130419
17	DEPRESSION/	139209

18	(predictor* or "risk factor*" or determinant* or "sexual behaviour" or "multiple sex partner*" or extramarital* or "sell* sex*" or "transactional sex" or prostitut* or "sex work" or condom* or "unsafe sex" or "unprotected sex" or knowledge or "social influenc*" or "peer influenc*" or "social norm" or cultur* or socioeconomic* or "social environmental*" or socioenvironment* or Stigma or discriminat* or "Psychological impact" or "Social impact" or education or "school attendance" or "withdraw* from school" or Stress or "Psychosocial impact" or stress or depression or distress).tw,kf.	5029349
19	or/10-18	5299336
20	family/ or family relations/	92433
21	(family* or families).tw,kf.	1073772
22	20 or 21	1106485
23	Developing Countries/	79007
24	((Developing or Less developed or low resource* or disadvantaged or resource limited or poor or low* or middle income*) adj (countr* or region* or nation? or area*)).tw,kf.	137093
25	23 or 24	169922
26	4 and 9 and 19 and 22 and 25	1254
27	limit 26 to (english language and yr="2004 - 2021")	226

Database: SCOPUS

Search strategy:

TITLE-ABS-KEY ((hiv* OR "Human immunodeficiency virus" OR aids)) AND TITLE-ABS-KEY ((wives OR wife OR mothers OR female* OR girl* OR women OR woman)) AND TITLE-ABS-KEY ((predictor* OR "risk factor*" OR determinant* OR "sexual behaviour" OR "multiple sex partner*" OR extramarital* OR "sell* sex*" OR "transactional sex" OR prostitut* OR "sex work" OR condom* OR "unsafe sex" OR "unprotected sex" OR knowledge OR "social influenc*" OR "peer influenc*" OR "social norm*" OR cultur* OR sociocultural* OR socioeconomic* OR "social environmental*" OR socioenvironment* OR stigma OR discriminat* OR "psychological impact" OR "social impact" OR stress OR distress OR depression OR "psychosocial impact")) AND TITLE-ABS-KEY ((family* OR families)) AND TITLE-ABS-KEY (((developing OR "Less developed" OR "low resource*" OR disadvantaged OR "resource limited" OR poor OR "low* OR middle income*") W/0 (countr* OR region* OR nation? OR area*))) AND (LIMIT-TO (PUBYEAR, 2021) OR LIMIT-TO (PUBYEAR, 2004)) AND (LIMIT-TO (LANGUAGE, "English")) <br>Results: 30

Database(s): ProQuest

Search strategy:

noft((HIV* OR "Human immunodeficiency virus" OR AIDS) AND (Wives OR Wife OR Mothers OR female* OR girl* OR wom?n) AND (predictor* OR "risk factor*" OR determinant* OR "sexual behaviour" OR "multiple sex partner*" OR "sell* sex*" OR "transactional sex" OR prostitut* OR "sex work" OR condom* OR "unsafe sex" OR "unprotected sex" OR knowledge OR "social influenc*" OR "peer influenc*" OR "social norm" OR cultur* OR sociocultural* OR socioeconomic* OR "social environmental*" OR socioenvironment* OR Stigma OR discriminat* OR "Psychological impact" OR "Social impact" OR "Psychosocial impact" OR distress OR stress

OR Depression) AND (family* OR families) AND ((Developing OR "Less developed" OR "low resource*" OR disadvantaged OR "resource limited" OR poor OR "low* OR middle income*") NEAR/0 (countr* OR region* OR nation? OR area*))) AND PEER(yes)<br><br>Results: 272

Supplementary Materials S2: JBI Critical Appraisal instruments

JBI Critical Appraisal Checklist for Qualitative Research

Reviewer_____________________________Date_____________________

Author ______________________________Year_______Record Number_____

	Yes	No	Unclear	Not applicable
1. Is there congruity between the stated philosophical perspective and the research methodology?	☐	☐	☐	☐
2. Is there congruity between the research methodology and the research question or objectives?	☐	☐	☐	☐
3. Is there congruity between the research methodology and the methods used to collect data?	☐	☐	☐	☐
4. Is there congruity between the research methodology and the representation and analysis of data?	☐	☐	☐	☐
5. Is there congruity between the research methodology and the interpretation of results?	☐	☐	☐	☐
6. Is there a statement locating the researcher culturally or theoretically?	☐	☐	☐	☐
7. Is the influence of the researcher on the research, and vice- versa, addressed?	☐	☐	☐	☐
8. Are participants, and their voices, adequately represented?	☐	☐	☐	☐
9. Is the research ethical according to current criteria or, for recent studies, and is there evidence of ethical approval by an appropriate body?	☐	☐	☐	☐
10. Do the conclusions drawn in the research report flow from the analysis, or interpretation, of the data?	☐	☐	☐	☐

Overall appraisal: Include ☐ Exclude ☐ Seek further info ☐

Comments (Including reason for exclusion)

____

____

____

JBI Critical Appraisal Checklist for Analytical Cross Sectional Studies

Reviewer_____________________________ Date_____________________

Author _____________________________ Year_______ Record Number_____

	Yes	No	Unclear	Not applicable
1. Were the criteria for inclusion in the sample clearly defined?	☐	☐	☐	☐
2. Were the study subjects and the setting described in detail?	☐	☐	☐	☐
3. Was the exposure measured in a valid and reliable way?	☐	☐	☐	☐
4. Were objective, standard criteria used for measurement of the condition?	☐	☐	☐	☐
5. Were confounding factors identified?	☐	☐	☐	☐
6. Were strategies to deal with confounding factors stated?	☐	☐	☐	☐
7. Were the outcomes measured in a valid and reliable way?	☐	☐	☐	☐
8. Was appropriate statistical analysis used?	☐	☐	☐	☐

Overall appraisal: Include ☐ Exclude ☐ Seek further info ☐

Comments (Including reason for exclusion)

____

____

____

JBI Critical Appraisal Checklist for Case Reports

Reviewer__________________________________Date__________________________

Author ___________________________________Year________Record Number______

	Yes	No	Unclear	Not applicable
1. Were patient's demographic characteristics clearly described?	☐	☐	☐	☐
2. Was the patient's history clearly described and presented as a timeline?	☐	☐	☐	☐
3. Was the current clinical condition of the patient on presentation clearly described?	☐	☐	☐	☐
4. Were diagnostic tests or assessment methods and the results clearly described?	☐	☐	☐	☐
5. Was the intervention(s) or treatment procedure(s) clearly described?	☐	☐	☐	☐
6. Was the post-intervention clinical condition clearly described?	☐	☐	☐	☐
7. Were adverse events (harms) or unanticipated events identified and described?	☐	☐	☐	☐
8. Does the case report provide takeaway lessons?	☐	☐	☐	☐

Overall appraisal: Include ☐ Exclude ☐ Seek further info ☐

Comments (Including reason for exclusion)

____

____

____

Supplementary Materials S3: Assessment of methodological quality (n=17)

**Authors**	**Q1**	**Q2**	**Q3**	**Q4**	**Q5**	**Q6**	**Q7**	**Q8**	**Q9**	**Q10**	**%**
Azhar	Y	Y	Y	Y	Y	Y	U	Y	Y	Y	100
Chi et al	Y	Y	Y	Y	Y	N	Y	Y	Y	Y	90
de Souza	Y	Y	Y	Y	Y	Y	Y	Y	N	Y	90
Fauk et al	Y	Y	Y	Y	Y	Y	Y	Y	Y	Y	100
Halli et al	Y	Y	Y	Y	Y	Y	Y	Y			100
Halimatusa'diah	Y	Y	Y	Y	Y	Y	Y	Y	N	Y	90
Ismail et al	Y	Y	Y	Y	Y	Y	Y	Y	Y	Y	100
Liamputtong et al	Y	Y	Y	Y	Y	Y	Y	Y	Y	Y	100
Mathew et al	Y	Y	Y	Y	Y	Y	U	Y	Y	Y	100
Nguyen et al	Y	Y	Y	Y	Y	N	Y	Y	Y	Y	90
Paxton et al	Y	Y	Y	Y	Y	Y	U	Y			100
Qin et al	Y	Y	Y	Y	Y	U	Y	Y			100
Srivastava et al	Y	Y	Y	Y	Y	Y	Y	Y	Y	Y	100
Subramaniyan et al	Y	Y	Y	Y	Y	N	U	Y	Y	Y	89
Thomas et al	Y	Y	Y	Y	Y	Y	Y	Y	Y	Y	100
Zang et al	Y	Y	Y	Y	Y	Y	Y	Y			100
Yang et al	Y	Y	Y	Y	Y	N	U	Y	Y	Y	89

Q= Question; Y= Yes; N= No; U= Unclear
